# Thermal-Driven Formation of Silver Clusters Inside Na/Li FAUY Zeolites for Formaldehyde Detection

**DOI:** 10.3390/nano12183215

**Published:** 2022-09-16

**Authors:** Jianzhong Yu, Song Ye, Xinling Xv, Ling Pan, Peixuan Lin, Huazhen Liao, Deping Wang

**Affiliations:** School of Materials Science and Engineering, Tongji University, Shanghai 201804, China

**Keywords:** FAUY zeolite, silver clusters, luminescence property, formaldehyde detection, auger parameter

## Abstract

In this research, the LiY zeolite was firstly synthesized by using NaY as the parent zeolite; thereafter, the LiYAg and NaYAg zeolites created for formaldehyde gas detection were prepared with further Ag^+^-Li^+^/Na^+^ exchange and a mild thermal treatment at 300 °C to promote the formation of luminescent Ag CLs. The spectra experimental results indicated that Ag CLs showed stronger and blue-shifted emissions in LiYAg compared with in NaYAg, and the emission intensity of Ag CLs in both zeolites monotonously decreased when exposed to increasing formaldehyde gas content. Moreover, the linear dependence of the Ag CLs’ emission intensity variation on formaldehyde content indicated a reliable method for fast and sensitive formaldehyde detection. According to the XPS, UV–vis absorption, and N_2_ adsorption–desorption isotherm studies, the formaldehyde-gas-induced luminescence quenching of Ag CLs is due to the formation of Ag_2_O and Ag NPs, in which the higher content of Ag^+^/Ag^0^ redox couples in LiYAg and larger surface area of NaYAg benefit the precise detection of formaldehyde gas in low- and high-content ranges, respectively. Furthermore, the blue-shifted peak position and widened FWHM of Ag CLs can also be used for the indication of formaldehyde gas and the detection limit of NaYAg and LiYAg, which both meet with the standards of the WHO and OSHA.

## 1. Introduction

Noble metal clusters consisting of only a few atoms or ions exhibit outstanding catalytic and optical properties due to their molecule-like structures and energetics, which are quite different from their nanoparticle and bulk metal counterparts [1,2,3]. Recent studies suggest that the silver clusters (Ag CLs) possess intriguing luminescence properties, with emissions covering the visible range and external quantum efficiency up to 97%, which is mainly governed by the clusters’ sizes, charges, and geometries [4,5,6]. However, precise control of Ag CLs’ luminescence properties remains challenging due to their tendency to aggregate into larger particles to reduce surface energy, thus irreversibly losing their emissive abilities [7,8,9]. Various host materials and synthesis approaches have been applied so far to stabilize Ag CLs of various sizes and chemical states, such as using DNA, polymers, rigid glasses, and zeolites [8,10,11].

Zeolites are porous materials with molecular-sized channels and cages that act as promising scaffolds for luminescent Ag CLs to avoid aggregating through spatial restrictions [3,9,12]. Benefitting from the diverse framework topologies, high cation exchange rates, tunable Si/Al ratios, and high thermal stabilities, the Ag^+^ can be readily loaded into zeolites and the luminescence properties of Ag CLs formed through subsequent external driven energy can be flexibly manipulated for diverse applications, such as white LEDs, imaging, and sensors [6,7,13,14]. It was reported that the emission peak wavelengths of Ag CLs in FAU zeolites are Si/Al ratio, extra framework cation, and thermal treatment condition dependent, and can be turned from 460 to 560 nm [7,15,16,17]. Further comparisons of the luminescence properties of Ag CLs formed with X-ray irradiation and thermal treatment indicate that the latter promotes the formation of luminescent clusters with specific small nuclearities, while the former produces a wide distribution of cluster species and larger nanoparticles [18,19]. Therefore, thermal treatment has been commonly used to promote the even formation of Ag CLs, and recent studies demonstrated that thermal treatment temperature influences the water content and stability of Ag CLs in LAT and FAU zeolites [6,20]. Furthermore, the thermal treatment atmosphere also manipulates the formation of Ag CLs in FAUX zeolites, the smaller Ag CLs (Ag_2_^2+^, Ag_3_^+^, and Ag_3_^2+^) can be formed under the oxygen flow, while the larger Ag CLs (Ag_4_*^n^*^+^ and Ag_8_*^m^*^+^) can be formed under a hydrogen atmosphere [21,22]. Additionally, extra framework modifications through Li^+^-Na^+^ exchange before Ag^+^ loading have proved to be ideal for improving the stability and luminescence intensity of Ag CLs because of the small radius and high affinity of Li^+^ to coordinate with zeolite framework oxygen [6,16,17].

The volatile organic solvent formaldehyde (HCHO) is a major indoor pollutant released from buildings and decorative materials that is detrimental to human health and may cause serious organ damage and even central nervous system injury [23,24,25]. The World Health Organization (WHO) and Occupational Safety and Health Administration (OSHA) have established that the long-term exposure-level limits for formaldehyde are 0.08 and 0.75 ppm, respectively [20,26]. Current formaldehyde detection methods include liquid/gas chromatography, spectroscopy, colorimetry, and electrochemical sensing; however, the practical application of these methods remains a big challenge because they are either highly cost- or time-consuming, involved in harmful reagents, or hard to achieve with high efficiency and sensitivity [20,27,28,29]. Fast-response, high-accuracy, and low-content formaldehyde-detection approaches are still under development.

In this study, faujasite zeolite with a high ion-exchange rate was used as a scaffold for Ag NCs to achieve a bright emission under UV excitation. The LiY zeolite was firstly synthesized by using NaY as the parent zeolite; thereafter, the LiYAg and NaYAg zeolites used for formaldehyde gas detection were prepared with a further Ag^+^-Li^+^/Na^+^ exchange and mild thermal treatment at 300 °C to promote the formation of luminescent Ag CLs. Under UV excitation, the Ag CLs showed stronger and blue-shifted emissions in LiYAg compared with in NaYAg, and the emission intensities of Ag CLs in both zeolites decreased when exposed to formaldehyde gas. The XPS, UV–vis absorption, and N_2_ adsorption–desorption isotherm results indicated that the formaldehyde-gas-induced luminescence quenching is due to the formation of Ag_2_O and Ag NP. The linear fitting between the formaldehyde concentration and the emission intensity variations of Ag CLs indicated LiYAg and NaYAg can be used for the precise detection of formaldehyde in a wide range.

## 2. Materials and Methods

### 2.1. Materials and Reagents

We purchased commercially available zeolite Na-FAUY ((Na_6.5_^+^)[Al_6.5_Si_17.5_O_48_], denoted as NaY) with SiO_2_/Al_2_O_3_ = 5.1 in powder form from Alfa Aesar. We obtained LiNO_3_ (99.99%, metals basis) from Aladdin Chemical Co., Ltd., Shanghai, China. We purchased AgNO_3_ (99.8%) from Sinopharm Chemical Reagent Co., Ltd., Shanghai, China. We obtained formaldehyde (HCHO, 37–40 wt% in water, AR) from Tansoole, Shanghai, China. We used all the materials and reagents as received without any further purification and dissolved all the aqueous solutions in deionized water.

### 2.2. Preparation of LiY Zeolite

The LiY zeolites were obtain from Li^+^-exchange on parent NaY zeolites. Each 1 g NaY zeolite was suspended in a highly concentrated 50 mL LiNO_3_ aqueous solution (5 mol/L). The suspension was agitated for 24 h at 80 °C, and then recovered by centrifugation, washed with deionized water several times and dried overnight at 80 °C.

### 2.3. Preparation of Ag CLs-Loaded NaY and LiY Zeolites

We obtained Ag CLs-loaded NaY and LiY zeolites by Ag^+^ exchange on NaY and LiY zeolites and a subsequent calcination procedure. Firstly, we suspended each 1 g NaY and LiY zeolite in 50 mL AgNO_3_ aqueous solution (30 mmol/L) and then agitated for 1.5 h at 40 °C in dark. Subsequently, we recovered the suspension by centrifugation, washed with deionized water several times, and dried overnight at 80 °C. Finally, we calcined the resultant Ag^+^-exchanged NaY and LiY zeolites at 300 °C for 2 h in air and sealed in dried containers in dark for further analysis. We denoted the final products as NaYAg and LiYAg, respectively.

### 2.4. Formaldehyde Detection

The climate chamber for formaldehyde detection is self-made and is shown Figure S1. Firstly, we diluted 0.3 mL formaldehyde aqueous solution to 2000, 1000, 250, and 100 mL, corresponding to the actual concentrations of 0.05, 0.10, 0.40, and 1.00 mg/1000 L, respectively. We dropped each concentration of 10 μL formaldehyde aqueous solution into glass-surface vessel and then put it into the polytetrafluoroethylene (PTFE) container; meanwhile, we placed 0.5 g LiYAg and 0.5 g NaYAg powders in two separated crucibles and covered before putting into the same PTFE container. After evaporating for 2 h at 25 °C, the formaldehyde concentration in the PTFE container reaches a plateau, and then we removed the cover to expose LiYAg and NaYAg powders to formaldehyde gas for 30 min. We monitored the formaldehyde concentrations in the container by commercial test strip at the same time. We denoted the samples explored in formaldehyde gases as M-*x*, M = LiYAg, or NaYAg, *x* = 0.05, 0.10, 0.40, and 1.00 mg/1000L, respectively, according to the formaldehyde aqueous solution concentration. We denoted the LiYAg and NaYAg exposed to formaldehyde aqueous solution without dilution as LiYAg-Excess and NaYAg-Excess, respectively. For consistency, we also exposed the NaYAg and LiYAg in non-formaldehyde atmosphere for 30 min in the PTFE container and labeled them as LiYAg-Original and NaYAg-Original, respectively.

### 2.5. Measurements

We carried out the Raman spectra on HR Evolution, Longjumeau, France, scanned in the range of 200–900 cm^−1^. We recorded the X-ray diffraction (XRD) patterns on Rigaku Smartlab9 diffractometer, Tokyo, Japan, with Cu-K*α* radiation (*λ* = 1.5406 Å). We measured the photoluminescence (PL) and the photoluminescence of excitation (PLE) spectra with Edinburgh FLS980, Edinburgh, UK. We carried out the X-ray photoelectron spectroscopy (XPS) measurements on Thermo ESCALAB 250XI Waltham, MA, USA, by using Al *Kα* (*hυ* = 1486.6 eV, 650 μm of beam spot) as the incident radiation source, and we used the electron flood gun to minimize surface charging effect. We detected the high-resolution XPS spectra of Ag 3d with 30 eV pass energy and the Ag Auger spectra with 40 eV pass energy. We measured the UV–vis absorbance spectra in the wavelength range of 200–700 nm on Shimadzu UV-3600i Plus spectrophotometer, Kyoto, Japan, at room temperature and applied attenuators to remove the effects of background and noise. We measured N_2_ adsorption–desorption isotherm by Micromeritics ASAP2020, Atlanta, GA, USA, at 77 K and calculated the total surface area by Brunauer–Emmett–Teller (BET) method.

## 3. Results and Discussion

### 3.1. Structural and Chemical Analysis

**Figure 1:**
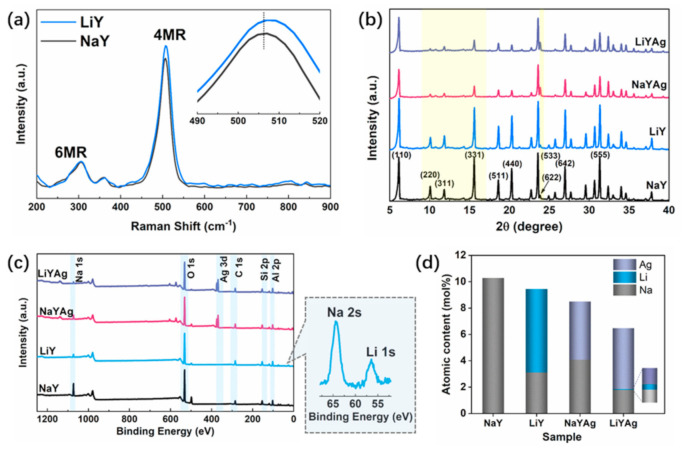
Raman spectra of LiY and NaY (**a**), XRD patterns (**b**), XPS survey spectra (**c**), and Na, Li, and Ag atomic contents (**d**) of NaY, LiY, NaYAg, and LiYAg, respectively.

**Figure 2:**
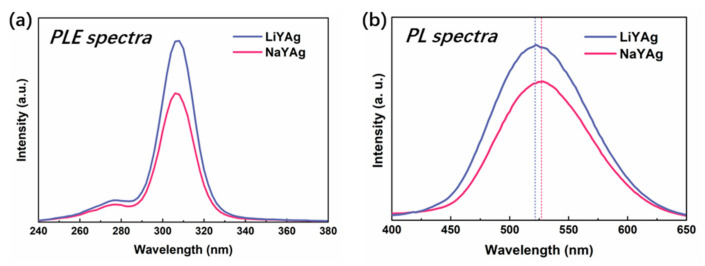
PLE (**a**) and PL (**b**) spectra of Ag CLs in LiYAg and NaYAg, respectively, under 306 nm excitation; inset: corresponding normalized PL spectra.

Charge-balancing cations coordinated to the zeolite framework ions can modify framework vibrations through electrostatic interaction. Figure 1 shows the Raman spectra of LiY and NaY have two main bands located at 360 and 506 cm^−1^, which can be attributed to the bending vibration of the six- (*δ* 6MR) and four-membered ring (*δ* 4MR), respectively. The high frenquency shift of the *δ* 4MR for LiY is caused by the increased T-O-T bonding force and reduced T-O-T angle brought by the substitution of Li^+^ for Na^+^ [30,31]. To study the effects of ion exchange on the phase purity and structure of the parent zeolite, the XRD patterns of LiY, NaYAg, and LiYAg are compared with that of NaY in Figure 2b. LiY, NaYAg, and LiYAg all maintain the structure of the parent NaY, while NaYAg and LiYAg show decreased diffraction intensities due to the later thermal treatment [8,32]. It is noticed that the diffraction peak intensities of the parent NaY zeolite (*I*) follow *I*_331_ > *I*_220_ > *I*_311_, which changed to *I*_331_ > *I*_311_ > *I*_220_ for LiY, NaYAg, and LiYAg. As the peaks of the (331), (311), and (220) facets are colsely related to the locations of cations in zeolites, this relative diffraction peak intensity varition can be attributed to the redistribution of the intra-zeolite charge-balancing cations of Na^+^, Li^+^, and Ag^+^ [33,34]. Furthermore, the increased diffraction intensity of the (622) facet in NaYAg and LiYAg also indicates the succssful incorporation of Ag^+^ [8,17].

The XPS survey spectra of NaY, LiY, NaYAg, and LiYAg are shown in Figure 1c, from which the Al 2p, Si 2p, C 1s, O 1s, and Na 1s peaks can be observed for all the samples, and the Li 1s and Ag 3d peaks can be observed in LiY, LiYAg, and NaYAg. The XPS chemical composition in Figure 1d shows that Na content drastically decreased from 10.29 atomic% in NaY to 3.12 atomic% in LiY due to the successful introduction of 6.32 atomic% Li. It is noticed that after Ag^+^ exchanged under the same conditions, the Ag content in NaYAg and LiYAg had different values of 4.41 and 4.63 atomic%, respectively. The different Ag content in NaYAg and LiYAg indicates the exchange efficiency of Ag^+^ is influenced by extra-framework cations, and the Li^+^-Ag^+^ has a higher exchange rate than Na^+^-Ag^+^, which has also been reported in previous studies [16,17].

### 3.2. Luminescence Property and Chemical State of Ag CLs

It is generally agreed that the stable Ag_3_*^n^*^+^ clusters can be formed inside the D6r cages of Ag^+^-exchanged FAUY zeolites after high-temperature thermal treatment at around 600 °C, which gives off bright emissions under UV excitation [18,35,36]. In this study, a low temperature of 300 °C was carried out to promote the formation of luminescent Ag CLs in the Ag^+^-exchanged NaY and LiY zeolites. The PLE and PL spectra of LiYAg and NaYAg in Figure 2 both show a strong excitation band peaked at 306 nm together with a very weak shoulder located at 265 nm, and a very broad emission band in the range of 450–600 nm, indicating the formation of Ag_3_*^n^*^+^ clusters [4,7,8]. Previous studies indicated that the Ag CLs formed under mild thermal treatment tended to be located outside the cavities and cages of FAUY zeolite, making them less stable with the surrounding atmosphere [20]. It is noticed that the emission intensity of Ag CLs is stronger in LiYAg than in NaYAg. This is because Ag content is higher in LiYAg than in NaYAg and, more importantly, Li^+^ can make Ag^+^ move to appropriate sites easily to form Ag CLs owing to its smaller radius than Na^+^, which promotes the formation and stabilization of Ag CLs due to their high affinity to coordinate with framework oxygen [6,16,17]. Moreover, the emission peak wavelength of Ag CLs slightly blue-shifted from 526 nm in NaYAg to 522 nm in LiYAg, which can be attributed to the reduced lattice parameter after the replacement of Na^+^ by Li^+^ [17,30].

**Figure 3:**
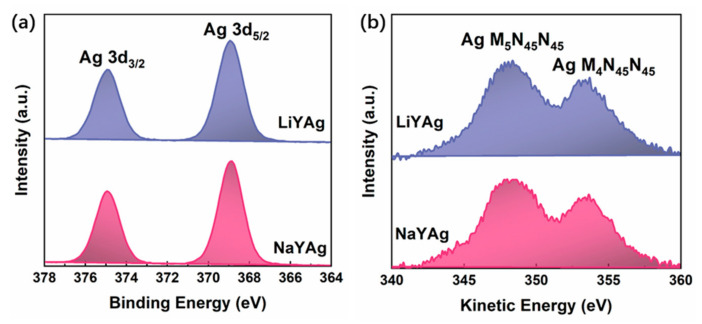
High-resolution XPS spectra of Ag 3d (**a**) and background-corrected Auger spectra of Ag MNN (**b**) in LiYAg and NaYAg, respectively.

Furthermore, the XPS measurement was carried out to identify the chemical state of silver species in LiYAg and NaYAg. The high-resolution XPS spectra of Ag 3d in Figure 3a presents two photoelectron peaks attributed to Ag 3d_5/2_ and Ag 3d_3/2_. Because of the small chemical shift and the binding energy overlap of Ag 3d in different silver species [1,37], the high-resolution XPS spectra of Ag 3d show no difference for LiYAg and NaYAg. Figure 3b shows Ag M_4_N_45_N_45_ Auger spectra, in which the two bands with discriminable kinetic energies for LiYAg and NaYAg can be attributed to Ag M_4_N_45_N_45_ and Ag M_5_N_45_N_45_, respectively (see Table S1). Therefore, the Auger parameter of Ag 3d, which is more sensitive to the chemical bonding environment, was calculated through the sum of the binding energy of Ag 3d_5/2_ and the kinetic energy of Ag M_4_N_45_N_45_ (Auger parameter = Ag 3d_5/2_ + Ag M_4_N_45_N_45_) [19,36,37], which are 722.3 and 722.5 eV for LiYAg and NaYAg, respectively. The small chemical shift compared with Ag_2_O (724.1 eV) and the large chemical shift compared with Ag NPs (726.0 eV) and metallic silver indicate the formation of Ag CLs in both zeolites, while the higher Auger parameter of Ag MNN in NaYAg than in LiYAg can be ascribed to the different extra-frame cations in the host zeolite [17,19,36,37].

### 3.3. Formaldehyde Sensor and Luminescence Quenching Mechanism

**Figure 4:**
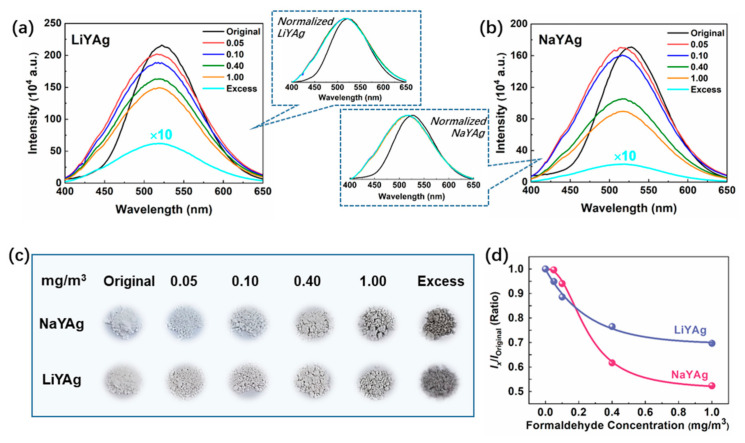
PL spectra of LiYAg (**a**) and NaYAg (**b**) before and after exposing to different contents of formaldehyde atmospheres; digital photographs of LiYAg and NaYAg after exposing to different contents of formaldehyde atmospheres under natural light (**c**); relative emission intensity of LiYAg and NaYAg at increasing contents of formaldehyde gases (**d**).

Figure 4a,b show the PL spectra of Ag CLs in LiYAg and NaYAg after exposing to different contents of formaldehyde gases. It can be seen that the emission intensity of Ag CLs in LiYAg and NaYAg both monotonously decrease with increasing-content formaldehyde gas, and the luminescence quenching on the whole is much more evident for NaYAg, which is in accordance with the results of previous research that the Ag CLs were more stable in Li-zeolite than in Na-zeolite [6,16,38]. It is worth mentioning that once exposed to formaldehyde gases, even a low concentration of 0.05 mg/m^3^ (lower than the limited permissible concentrations of WHO and OSHA), the Ag CLs show blue-shifted emissions and increased full width at half-maximum (FWHM) in both LiYAg and NaYAg, indicating the sensitive detection of very-low-content formaldehyde. The digital photos of NaYAg and LiYAg zeolite powders before and after being exposed to various contents of formaldehyde gases under natural light are shown in Figure 4c. The colors of the LiYAg and NaYAg powders both become dark with increasing-content formaldehyde gas, which is in line with the PL results.

According to the PL spectra in Figure 4a, the relationships between formaldehyde concentration (*x*) and the emission intensity variation (*y* = I_x_/I_original_) of Ag CLs in LiYAg and NaYAg were plotted in Figure 4d, which can be linearly fitted with Equations (1) and (2).

(1) $$y=A_{1}\times e^{\frac{-x}{t_{0}}}+A_{2}$$

where *A*_1_ = 0.30, *A*_2_ = 0.69, *t*_0_ = 0.25, *R*^2^ = 0.991.

(2) $$y=\frac{B_{2}-B_{1}}{\left[1+{\left(\frac{x}{x_{0}}\right)}^{p}\right]}+B_{2}$$

where *B*_1_ = 1.00, *B*_2_ = 0.51, *x*_0_ = 0.25, *p* = 2.43, *R*^2^ = 0.998;

The first derivative is bigger for the fitting curve from Equation (1) than the curve from Equation (2) when *x* < 0.18 mg/m^3^, indicating that LiYAg can more sensitively detect low-content formaldehyde than NaYAg. This can be ascribed to the higher Ag atomic content (See Figure 1d) or the increased quantity of Ag^+^/Ag^0^ redox couples that constitute Ag CLs in LiYAg. It is generally agreed that the Ag^+^/Ag^0^ redox couples can generate oxygen vacancies (${\;V}_{0}^{¨}$) through Equation (3), and the ${\;V}_{0}^{¨}$ can effectively promote the absorption of formaldehyde gas [8,26,39,40].

(3) $$4{\mathrm{Ag}}^{+}+2O^{2-}\to 4{\mathrm{Ag}}^{0}+O_{2}↑+{\;V}_{0}^{¨}$$

Interestingly, after exposing to high concentrations of formaldehyde gases, I_x_/I_original_ shows a more significant decrease in NaYAg than in LiYAg. In order to figure out the reason for the different spectra responses to high-content formaldehyde gas, the N_2_ adsorption–desorption isotherms of LiYAg and NaYAg were measured (See Figure S2), and we found both exhibited typical type I adsorption isotherm of microporous phase [41,42,43]. Application of the BET model confirms the surface area of LiYAg and NaYAg are 713.8 and 764.3 m^2^/g, respectively, indicating a higher adsorption capability of NaYAg than LiYAg. Therefore, NaYAg can adsorb more formaldehyde gases than LiYAg when exposed to high-content formaldehyde gases, resulting in more significantly decreased emission intensity. Therefore, the higher content of Ag^+^/Ag^0^ redox couples in LiYAg and larger surface area of NaYAg can benefit the precise detection of formaldehyde gas in low- and high-content ranges, respectively.

**Figure 5:**
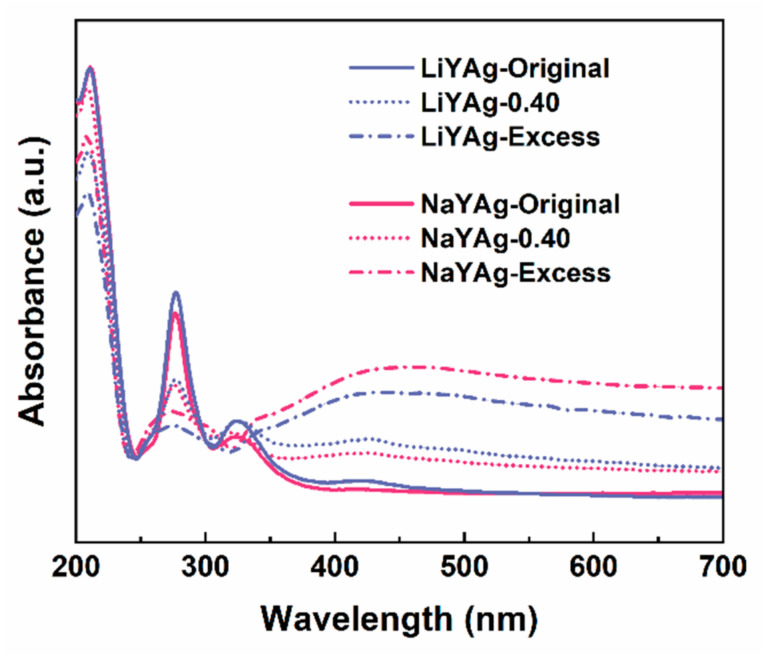
UV–vis absorbance spectra of LiYAg and NaYAg before and after exposing in different formaldehyde atmospheres.

To further investigate the mechanism that is responsible for the formaldehyde-gas-induced luminescence quenching of Ag CLs, the UV–vis absorbance spectra were firstly measured for LiYAg and NaYAg before and after exposing to 0.40 mg/m^3^ and excess formaldehyde gases. As shown Figure 5, the strong absorption band peaked at 213 nm, which can be ascribed to the 4d^10^→4d^9^5s^1^ electronic transition of isolated silver ions in FAU and LTA zeolites, and the absorptions located at 275 and 324 nm belonged to the Ag_3_*^n^*^+^ clusters confined in FAU zeolites [6,17,18]. It is noticed that the absorption intensity of Ag^+^ is very close for LiYAg and NaYAg, while that of Ag CLs is stronger in LiYAg than in NaYAg, indicating more Ag CLs were formed in LiYAg. After exposing to formaldehyde gas, the absorption intensity of Ag CLs rapidly decreased for both LiYAg-0.40 and NaYAg-0.40, and further reduced in LiYAg-Excess and NaYAg-Excess. This is in accordance with the PL spectra in Figure 4a,b. Moreover, the surface plasma resonance (SPR) absorption band located around 450 nm can be observed for NaYAg-Excess and LiYAg-Excess, indicating the formation of Ag NPs [7,8,17]. Therefore, we speculate that the exposure to formaldehyde gas reduced the luminescence center number through inducing the transformation from ionic Ag CLs to metallic Ag NPs, thus resulting in quenched emission intensities.

**Figure 6:**
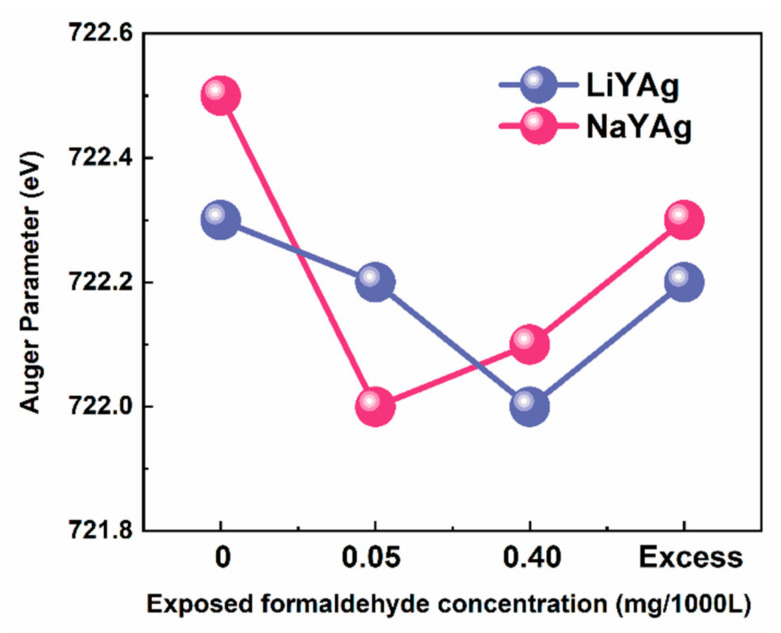
Auger parameter of Ag MNN in LiYAg and NaYAg before and after exposing in different formaldehyde-content atmospheres.

Based on the above considerations, it is necessary to identify the chemical state of silver before and after exposing to formaldehyde gas. According to the XPS data in Table S2, we calculated the Auger parameter of Ag in LiYAg and NaYAg before and after exposing to 0.05 and 0.40 mg/1000L, as well as excess formaldehyde gases. As shown in Figure 6, the Auger parameter of Ag in LiYAg-x and NaYAg-x firstly increases and then decreases after exposure to high-content formaldehyde, and the increase and decrease in Auger parameter indicate the reduction and oxidation of Ag CLs, respectively [32,37,44]. On the one hand, the Ag^+^ that constitutes Ag CLs can acquire electrons from framework oxygen, hydration water, and formaldehyde to form Ag^0^ and ${\;V}_{0}^{¨}$ through the above Equation (3) [8,15,45,46]. On the other hand, the Ag^0^ in Ag CLs can be oxidized to Ag_2_O through Equation (4) [20,47].

(4) $$4{\mathrm{Ag}}^{0}+O_{2}\to 2{\mathrm{Ag}}_{2}$$

Therefore, the Ag^+^/Ag^0^ redox couples can reach an eqiuilibrium through the reactions described in Equations (3) and (4) when exposed to formaldehyde gases. Under a low-content formaldehyde atmosphere, the oxidation of Ag^0^ into Ag^+^ through Equation (4) dominates, resulting in decreased Auger parameter; however, in high-content formaldehyde atmospheres, there are excessive formations of Ag^0^ through Equation (3) because Ag^+^ can acquire more electrons from O^2−^ in the adsorbed formaldehyde, leading to an increased Auger parameter. The excessively formed Ag^0^ can aggregate into large Ag NPs, which allows the further increase of the Auger parameter in accordance with the absorption in Figure 5. Consequently, the formaldehyde-gas-induced luminescence quenching of Ag CLs is due to the dynamic formation of Ag_2_O and Ag NPs, and the adsorbed formaldehyde can be oxidized into water molecules and carbon dioxide, as described in Equations (5) and (6) [20,26,48].

(5) $$\frac{1}{2}O_{2\;\left(\mathrm{gas}\right)}+{\;e}^{-}\;\to {\;O}_{\left(\mathrm{ads}\right)}^{-}$$

(6) $$\mathrm{HCHO}+2O_{\left(\mathrm{ads}\right)}^{-}\;\to {\;\mathrm{CO}}_{2}+{\;H}_{2}O\;+{\;e}^{-}$$

## 4. Conclusions

In conclusion, the LiY zeolite was firstly synthesized by using NaY as the mother zeolite; thereafter, the Ag CLs loaded LiYAg and NaYAg zeolites with mild thermal treatment were prepared. The spectra results indicated that Ag CLs show stronger and blue-shifted emissions in LiYAg compared with in NaYAg. The emission intensity of Ag CLs monotonously decreases when exposed to increasing contents of formaldehyde gas in both zeolites, and the linear fittings between the formaldehyde concentration and the emission intensity variations of Ag CLs were obtained for LiYAg and NaYAg. The XPS, UV–vis absorption, and N_2_ adsorption–desorption isotherm results indicated that the formaldehyde-gas-induced luminescence quenching of Ag CLs is due to the dynamic formation of Ag_2_O and Ag NPs, and the higher content of Ag^+^/Ag^0^ redox couples in LiYAg and the larger surface area of NaYAg benefit the precise detection of formaldehyde gas in low- and high-content ranges, respectively. Furthermore, the blue-shift peak position and widened Ag CLs FWHM can also be used for the indication of formaldehyde gas and the low detection limit of 0.05 mg/1000 L for NaYAg and LiYAg, which meet the WHO and OSHA standards.

## Data Availability

Not applicable.

## Supplementary Materials

The following supporting information can be downloaded at: https://www.mdpi.com/article/10.3390/nano12183215/s1, Figure S1: Schematic diagram of experimental installation for the detection of formaldehyde gas; Figure S2: N_2_ adsorption–desorption isotherms of NaYAg and LiYAg and the corresponding BET surface area; Table S1: Binding energies and Auger parameters of Ag in NaYAg and LiYAg; Table S2: Binding energies and Auger Parameters of Ag MNN in NaYAg and LiYAg before and after exposing to air and different contents of formaldehyde atmosphere.
