# Supplementary material for: Thermal-Driven Formation of Silver Clusters Inside Na/Li FAUY Zeolites for Formaldehyde Detection
Source: Nanomaterials (Basel). 2022 Sep 16;12(18):3215. doi: 10.3390/nano12183215 (PMC9503286; doi:10.3390/nano12183215)
Supplement: Supplementary file 1 [file nanomaterials-12-03215-s001.zip › nanomaterials-1877722-supplementary.pdf]

nanomaterials

MDPI

Supplementary Materials

Thermal-Driven Formation of Silver Clusters Inside Na/Li FAUY Zeolites for Formaldehyde Detection

Jianzhong Yu, Song Ye *, Xinling Xv, Ling Pan, Peixuan Lin, Huazhen Liao and Deping Wang

School of Materials Science and Engineering, Tongji University, Shanghai 201804, China
* Correspondence: yesong@tongji.edu.cn

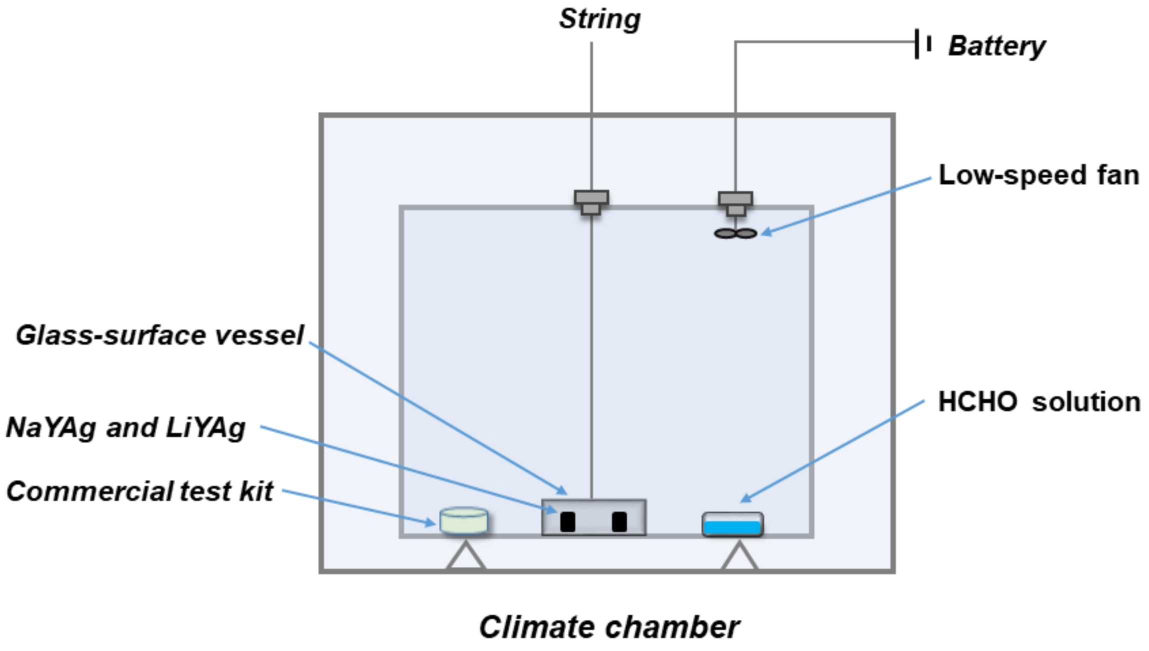

Figure S1. Schematic diagram of experimental installation for the detection of formaldehyde gas.

Table S1. Binding energies and Auger parameters of Ag in NaYAg and LiYAg.

Sample	Binding Energy Ag $3d_{5/2}$ (eV)	Kinetic Energy Ag $M_4N_{45}N_{45}$ (eV)	Auger Parameter (eV)
LiYAg	368.9	353.4	722.3
NaYAg	368.9	353.6	722.5

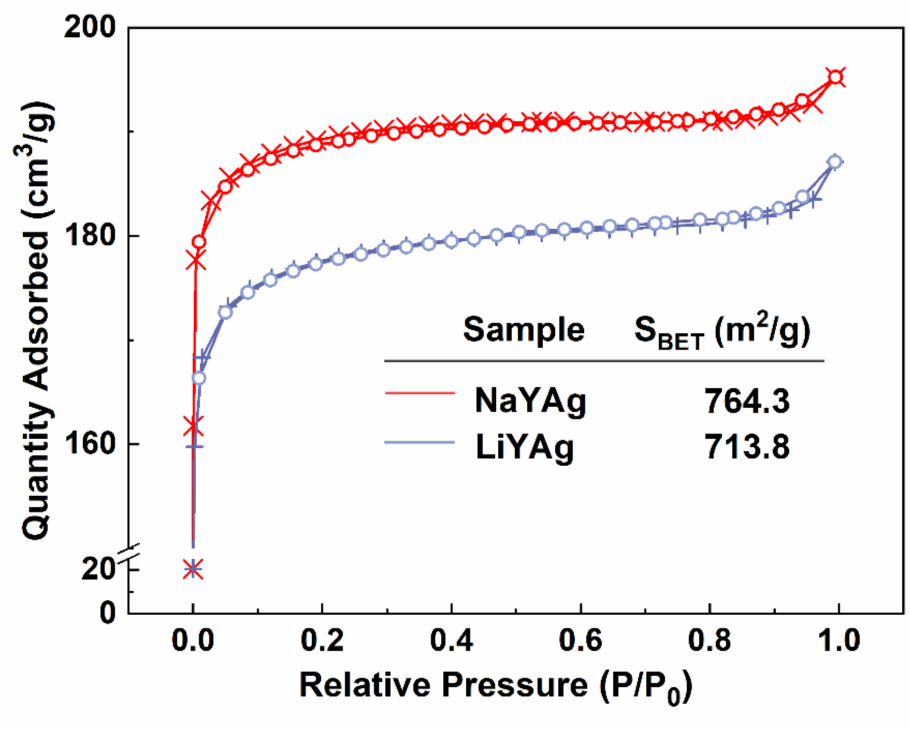

Figure S2. N_2 adsorption-desorption isotherms of NaYAg and LiYAg and the corresponding BET surface area.

Table S2. Binding energies and Auger Parameters of Ag MNN in NaYAg and LiYAg before and after exposing to air and different contents of formaldehyde atmosphere.

Sample	Binding Energy Ag $3d_{5/2}$ (eV)	Kinetic Energy Ag $M_4N_{45}N_{45}$ (eV)	Auger Parameter Ag $3d_{5/2}$+$M_4N_{45}N_{45}$ (eV)
LiYAg	368.9	353.4	722.3
LiYAg-0.05	368.9	353.3	722.2
LiYAg-0.40	368.9	353.1	722.0
LiYAg-Excess	368.8	353.4	722.2
NaYAg	368.9	353.6	722.5
NaYAg-0.05	368.9	353.1	722.0
NaYAg-0.40	368.9	353.2	722.1
NaYAg-Excess	369.0	353.4	722.3
